# Association of Nonprofit Hospitals’ Charitable Activities With Unreimbursed Medicaid Care After Medicaid Expansion

**DOI:** 10.1001/jamanetworkopen.2020.0012

**Published:** 2020-02-26

**Authors:** Charles Stoecker, Mollye Demosthenidy, Yixue Shao, Hugh Long

**Affiliations:** 1 Department of Health Policy and Management, Tulane University School of Public Health and Tropical Medicine, New Orleans, Louisiana

## Abstract

**Importance:**

In the United States, nonprofit hospitals receive tax-exempt status with the expectation that they provide a high level of benefit to local communities. Prior work has shown that Medicaid expansion reduced hospital spending on uncompensated care.

**Objective:**

To measure the association of tax-exempt hospital spending with community benefit and changes in uncompensated care after Medicaid expansion.

**Design, Setting, and Participants:**

This cohort study was performed using a difference-in-differences analysis (ie, a pre-post treatment-control design) to estimate changes in reported charitable categories associated with Medicaid expansion. Data from Internal Revenue Service form 990, Schedule H, tax filings for 2253 tax-exempt hospitals in the United States from 2012 to 2016 were used. Data were analyzed from June to November 2019.

**Exposure:**

The proportion of the hospital’s tax filing that spanned the period after Medicaid expansion.

**Main Outcomes and Measures:**

Hospital-reported spending on uncompensated care, unreimbursed Medicaid expenses, and other community benefit spending categories.

**Results:**

Across 2253 hospitals, mean (SD) uncompensated care costs between 2012 and 2016 were $4.20 million ($8.80 million) and unreimbursed Medicaid expenses were $7.60 million ($18.62 million). Compared with tax-exempt hospitals in states that did not expand Medicaid, those in states that did expand Medicaid reported mean reductions in their provision of uncompensated care of $1.11 million (95% CI, $0.35 million to $1.87 million; *P* < .001), representing a mean change of −2% (95% CI, −6% to 2%; *P* < .001). These reductions have been offset by mean reported increases in the provision of unreimbursed Medicaid expenses of $1.63 million (95% CI, $0.31 million to $2.94 million; *P* = .02), representing a mean increase of 2% (95% CI, 1% to 4%; *P* = .01). Tax-exempt hospitals in states that expanded Medicaid reported no statistically significant mean increase in spending on other community benefit activities.

**Conclusions and Relevance:**

In this study, large decreases in uncompensated care among tax-exempt hospitals associated with Medicaid expansion were not accompanied by increases in other reportable categories of community health benefit spending. Instead, they were accompanied by increased spending on unreimbursed Medicaid expenses.

## Key Points

**Question:**

Was Medicaid expansion associated with increased spending on community benefits by tax-exempt hospitals?

**Findings:**

In this cohort study of 2253 tax-exempt hospitals in the United States, Medicaid expansion was associated with 2% reported reductions in the provision of uncompensated care and 2% reported increases in the provision of unreimbursed Medicaid expenses.

**Meaning:**

Tax-exempt hospitals in states that expanded Medicaid did not substantially change community benefit spending, given that decreases in uncompensated care were offset by increases in unreimbursed Medicaid expenses.

## Teaser

This cohort study measures changes in tax-exempt hospital spending with community benefit and changes in uncompensated care after Medicaid expansion.

## Introduction

The federal government grants tax-exempt status to most nonprofit hospitals in the United States, foregoing approximately $24 billion in tax revenue in exchange for the provision of various benefits to the communities served by these organizations.^1,2^ Historically, most tax-exempt hospitals relied almost exclusively on the provision of uncompensated or charity care, rather than other community health investments, to demonstrate community benefit.^3^ A 2017 study^4^ found that, in 2009, tax-exempt hospitals spent approximately 8% of total operating expenses on community benefit, but most of that spending went toward charity care, unreimbursed Medicaid, and subsidized health services rather than community health improvement or community-building activities. Additional work found that, in the same year, tax-exempt hospitals spent a median of $130 per capita on community benefit activities, but only $11 of that total went toward community health improvement and community-building activities.^5^

In 2014, the Patient Protection and Affordable Care Act allowed states to expand their Medicaid programs to cover individuals earning up to 138% of the federal poverty level, resulting in a significant decrease in the number of uninsured individuals in those states. Consequently, tax-exempt hospitals located in expansion states saw mean decreases of $2.8 million per hospital in the reported cost of uncompensated care in their first year under expansion and mean increases of $3.2 million per hospital from Medicaid and Children’s Health Insurance Program revenue of all types.^6^

Given these reported reductions in the costs of uncompensated care, it is important to examine how tax-exempt hospitals’ spending on other community benefit activities has changed after Medicaid expansion. Prior work on this issue has examined how Medicaid expansion influenced spending on community benefit, broken down into patient care and community health benefits. Early evidence indicated that hospitals in expansion states were reporting declines in spending on uncompensated care while hospitals in nonexpansion states did not report changes.^7^

A 2018 study^8^ showed that tax-exempt hospitals did not increase their community benefit spending from 2012 to 2014, although another 2018 study^9^ found that teaching hospitals did increase overall community benefit spending from 2012 to 2015. Using hospital tax filings through 2014, Young et al^10^ found small decreases in charity care and little change in unreimbursed Medicaid programs as a proportion of hospital spending on community benefit. In follow-up work, Young et al^11^ found preliminary evidence that decreases in uncompensated care were partially offset by increases in unreimbursed Medicaid spending during the first year of Medicaid expansion. This study reexamined the question analyzed by Young et al^11^ with 2 additional years of data.

## Methods

Beginning in 2009, nonprofit hospitals were required to file Schedule H with their annual Internal Revenue Service form 990. This schedule details their net spending in 17 community benefit categories, as follows^12^: financial assistance at cost, unreimbursed Medicaid, cost of other means-tested government programs, community health improvement services and community benefit operations, health professions education, subsidized health services, research, cash and in-kind contributions for community benefit, physical improvements and housing, economic development, community support, environmental improvements, leadership development and training for community members, coalition building, community health improvement advocacy, workforce development, and other community-building activities. The first 2 categories (ie, financial assistance at cost and unreimbursed Medicaid expense) were the largest categories by reported dollar amount, and we focused on them in this study.

For this study, the universe of these filings from 2012 to 2016 was purchased in digitized format from GuideStar. This study linked Schedule H forms to data on the number of beds and teaching hospital status from the American Hospital Association. Because there are no common identifiers between the 2 data sets, this linkage was conducted in 3 steps. Data sets were first merged on hospital organization name, state, and city; then on state, city, and street address; and finally by manual review. Through this process, 10 300 of 12 103 Schedule H filings were matched, representing 2253 hospitals. The resulting data set was further linked with county-level information on demographic characteristics using the Surveillance, Epidemiology, and End Results database (National Cancer Institute) and economic information from the Regional Economic Information System (Bureau of Economic Analysis) (eTable 1 in the Supplement), which resulted in our final observation count of 10 154. We note that unmatched hospitals spent more on financial assistance at cost (and thus on total community benefit spending) (eTable 1 in the Supplement). This secondary data analysis was determined to be exempt from review and informed consent by the institutional review board of the Tulane University Human Subjects Office because there was no interaction with participants. This report follows the Strengthening the Reporting of Observational Studies in Epidemiology (STROBE) reporting guideline. Data were analyzed from June to November 2019.

### Statistical Analysis

To establish how hospitals’ tax filings on charitable activities changed after Medicaid expansion, this study used a generalized difference-in-differences regression model. Conceptually, the first difference was how each hospital’s spending pattern changed over time. The second difference was between these year-to-year differences for hospitals in states that expanded Medicaid and for those in states that did not. As states have expanded Medicaid in different years, the control group for the 2014 expansion consisted of states that have never expanded Medicaid and states that had not yet expanded Medicaid in 2014. For the states that expanded in 2015, the control group consisted of states that never expanded as well as those that had not yet expanded in 2015. We implemented this with a fixed-effects regression model using ordinary least squares, as follows: Expenditure_hcsy_ = α + βExpansion_sy_ + γ_s_ + δ_y_ + ϕX_scy_ + ε_hcsy_, in which Expenditure_hcsy_ denoted the reported expenditures in a charitable category by hospital *h* in county *c* in state *s* during the fiscal year ending in year *y*. The exposure variable, Expansion_sy_, indicated what proportion of a hospital’s fiscal year overlapped with Medicaid expansion. For example, a hospital with a fiscal year that closes on April 1 in a state that expanded Medicaid on January 1, 2014, was given a treatment value of 0.25 for the filing on April 1, 2014, because 25% of that fiscal year occurred after Medicaid expansion. Because the data set of tax filings used in this study ended in 2016, follow-up time necessarily varied according to both when a state enacted Medicaid expansion and when a hospital’s fiscal year ended. For a hospital with a fiscal year ending on December 31 in a state that passed Medicaid expansion on January 1, 2014, there were 2 complete tax filings after enactment. Hospitals in states with later expansions had more limited observations in the postenactment period.

Our model included state fixed effects to account for state differences in demographic characteristics and policy environments. It also included year fixed effects, based on the year the fiscal year ended, to account for secular trends in and national shocks to hospital finances. The econometric model controlled for county population, proportion of male residents, proportion of residents aged 65 years or older, proportion of black residents, proportion of Hispanic residents, income per capita, government transfers per capita, and employment per capita. We included county population for several reasons, including the fact that larger counties may have a more robust public health department that may mitigate some hospital expenditures or larger populations who use Medicaid or do not pay for care. We controlled for age and other demographic characteristics to adjust for any extent to which these characteristics may be associated with cost of uncompensated or unreimbursed Medicaid expenses. Government transfers per capita reflected how much total government spending flows into a county and served to correct for counties that receive government spending per person. To account for potential serial correlation in hospital finances, serial correlation in the state regulatory landscape, and correlations in hospital spending behavior within states, the model allowed for arbitrary correlation in standard errors within a state.

This study examined the evolution of the association of Medicaid expansion with hospital spending on charity care with an event study model, as follows: Expenditure_hcsy_ = α + β_1_Expansion_sy≤−2_ + β_2_Expansion_sy = 0_ + β_3_Expansion_sy = 1_ + β_4_Expansion_sy≥2_ + γ_s_ + δ_y_ + ϕX_scy_ + ^ε^hcsy. This equation was similar to the main estimation equation, but unpacked the Expansion_sy_ treatment variable from the main model into a set of time dummies. The first Expansion_sy ≤ -2_ term was an indicator variable that took the value 1 for fiscal filings from hospitals at least 2 years before a state expanded Medicaid and 0 otherwise. The second took the value 1 for fiscal filings from hospitals in the year a state expanded Medicaid and 0 otherwise, and so on. The omitted category was the year immediately before expansion. Thus, the coefficients on each of these variables gave the change between the indicated time period and the year immediately before expansion. To satisfy the parallel trends assumption, we expected there would be no statistical significance on the first coefficient (indicating there was no change associated with Medicaid expansion between the period at least 2 years before expansion and 1 year before expansion). Statistical significance in later coefficients indicated the timing of any association of expansion with the given expenditure category.

All data analysis was performed using Stata version 15.1 (StataCorp). We reported standard deviations of mean values and 95% CIs around regression coefficients using *t* tests. Statistical significance was set at *P* < .05, and all tests were 2-tailed.

## Results

**Figure 1.**
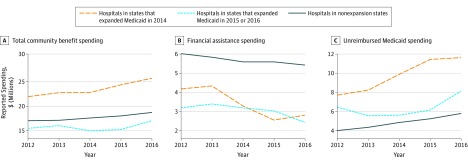
Benefit Spending by Tax-Exempt Hospitals in Expansion and Nonexpansion States by Year States are grouped according to those that did not expand Medicaid during the study period, those that expanded Medicaid in 2014, and those that expanded in 2015 or 2016.

Figure 1 plots unadjusted trends of the 3 key outcome variables (ie, total community benefit spending, financial assistance at cost, and unreimbursed Medicaid expenses) according to when states expanded Medicaid. In 2014, mean expenditures on total community benefit for hospitals in states that expanded Medicaid in 2014, hospitals in states that expanded Medicaid in 2015 and 2016, and hospitals that did not expand Medicaid between 2014 and 2016 were $22.8 million, $15.0 million, and $17.6 million, respectively. In 2016, they were $25.7 million, $17.1 million, and $18.7 million, respectively. Lines are roughly parallel between the states that did not expand Medicaid during our sample window and those that expanded in 2014. We turn to the event study graphs to assess whether these trends were parallel in the period before expansion.

**Figure 2.**
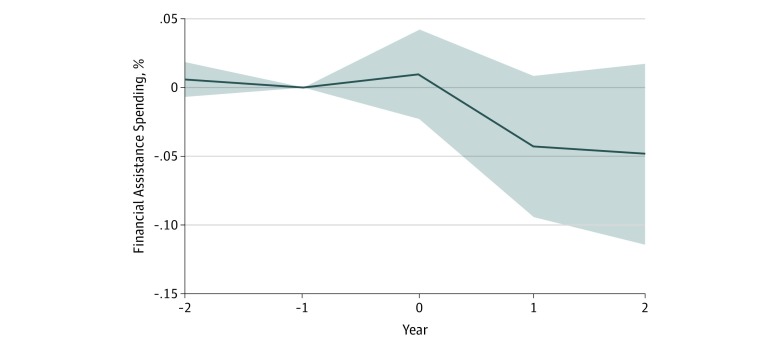
Dynamic Associations of Medicaid Expansion With Reported Financial Assistance at Cost by Tax-Exempt Hospitals Data came from hospital reports of charitable activities on tax filings from 2012 to 2016.

Medicaid expansion was associated with tax-exempt hospitals reporting decreased uncompensated or charity care, but this decrease was offset by increases in reported expenditures on unreimbursed Medicaid expenses. Among the 2253 hospitals examined in this study, mean (SD) spending on charity care (ie, free or reduced-price care given by hospitals to the uninsured) was $4.20 million ($8.80 million). Tax-exempt hospitals in states that expanded Medicaid reported mean reductions of $1.11 million (95% CI, $0.35 million to $1.87 million; *P* < .001), representing a mean change of −2% (95% CI, −6% to 2%; *P* < .38), in their provision of financial assistance spending compared with tax-exempt hospitals in states that did not expand Medicaid (Table 1). This was associated with reported changes in this expenditure category in filings 1 and 2 years after Medicaid expansion (year 0: β = 0.01; 95% CI, −0.02 to 0.04; year 1: β = −0.04; 95% CI, −0.09 to 0.01; year 2: β = −0.05; 95% CI, −0.11 to 0.02) (Figure 2). We found no contemporaneous decline in charity care for filings made within a year of Medicaid expansion. To assess whether these results may have been the consequence of nonparallel pretrends, we examined the coefficient 2 years before Medicaid expansion. Because this was not statistically significantly different from the omitted category (ie, 1 year before expansion), we found no evidence of nonparallel pretrends.

**Table 1.** Association of Medicaid Expansion With Community Benefit Activities by Tax-Exempt Hospitals From 10 154 Observations

Activity	DID (95% CI)	*P* Value	DID With Controls (95% CI)^a^	*P* Value
Total community benefit spending, millions of $	0.21 (−2.22 to 2.64)	.86	0.10 (−2.49 to 2.69)	.94
Proportion of total community benefit, %	<0.01 (−0.01 to 0.01)	.63	<0.01 (−0.00 to 0.01)	.61
Financial assistance spending, millions of $	−1.26 (−2.03 to −0.48)	<.001	−1.11 (−1.87 to −0.35)	<.001
Proportion of financial assistance spending, %	−0.02 (−0.06 to 0.02)	.35	−0.02 (−0.06 to 0.02)	.38
Unreimbursed Medicaid spending, millions of $	1.67 (0.33 to 3.02)	.02	1.63 (0.31 to 2.94)	.02
Proportion of unreimbursed Medicaid spending, %	0.02 (0.01 to 0.04)	.01	0.02 (0.01 to 0.04)	.01

Abbreviation: DID, difference-in-differences.

^a^ Adjusted with county-level controls for population, proportion of male residents, proportion of residents aged 65 years or older, proportion of black residents, proportion of Hispanic residents, income per capita, government transfers per capita, employment per capita, and state and year fixed effects.

**Figure 3.**
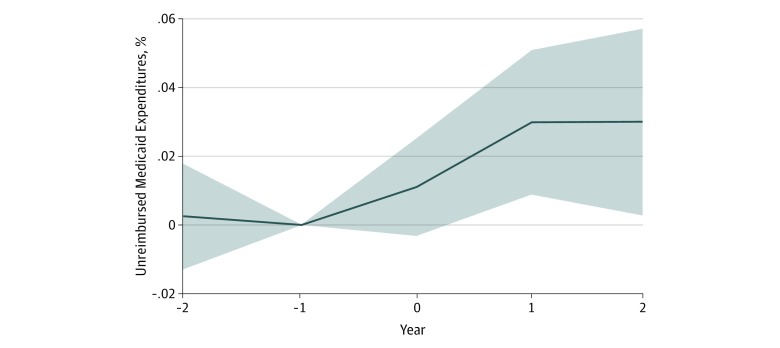
Dynamic Associations Between Medicaid Expansion With Reported Unreimbursed Medicaid Expenditures by Tax-Exempt Hospitals Data came from hospital reports of charitable activities on tax filings from 2012 to 2016.

Mean (SD) spending on unreimbursed Medicaid (ie, the difference between the mean cost allocated to Medicaid patients and Medicaid payments received by hospitals) was $7.60 million ($18.62 million). This amount increased by $1.63 million (95% CI, $0.31 million to $2.94 million; *P* = .02), representing a mean increase of 2% (95% CI, 1% to 4%; *P* = .01) among hospitals in states that expanded Medicaid compared with hospitals in states that did not expand Medicaid (Table 1). When we analyzed the timing of this change with an event study, there was no statistically significant contemporaneous association at year 0 (year 0: β = 0.01; 95% CI, −0.003 to 0.03; year 1: β = 0.03; 95% CI, 0.01 to 0.05; year 2: β = 0.03; 95% CI, 0.003 to 0.06) (Figure 3). The change appeared the year after states expanded Medicaid and was similar in the second year after expansion. Again, we found no evidence of nonparallel pretrends, given that there was no statistically significant difference between the association 2 years before Medicaid expansion and the omitted association 1 year before Medicaid expansion.

These 2 countervailing associations (ie, a decrease in reported spending on charity care and an increase in reported spending on unreimbursed Medicaid expenses) appear to have offset each other. Medicaid expansion was not associated with a statistically significant change in total community benefit spending. The point estimate was small compared with the 2 principal components at only $0.10 million (95% CI, −$2.49 million to $2.69 million) (Table 1).

We also examined the association of Medicaid expansion with the full array of Schedule H community benefit activities that are not related to charity care, Medicaid, or subsidized health expenses. The associations between changes in spending in these categories and Medicaid expansion were small; none were larger than $0.5 million, and none were statistically significant (Table 2; eTable 2 in the Supplement).

**Table 2.** Association Between Medicaid Expansion and Other Hospital Charitable Expenditures From 10 415 Observations

Category	DID, Mean (95% CI), Millions of $	*P* Value	DID With Controls, Mean (95% CI), Millions of $^a^	*P* Value
Costs of other means-tested government programs	−0.22 (−0.52 to 0.07)	.13	−0.20 (−0.48 to 0.07)	.14
Total charity care	0.20 (−1.44 to 1.85)	.80	0.32 (−1.31 to 1.95)	.69
Community health improvement services and community benefit operations	0.05 (−0.08 to 0.18)	.47	0.05 (−0.09 to 0.19)	.47
Health professions education	0.14 (−0.41 to 0.69)	.60	−0.10 (−0.68 to 0.48)	.72
Subsidized health services	0.15 (−0.18 to 0.48)	.36	0.19 (−0.12 to 0.49)	.23
Research	−0.28 (−1.24 to 0.67)	.55	−0.34 (−1.42 to 0.75)	.53
Cash and in-kind contributions to community groups	−0.02 (−0.19 to 0.15)	.81	0.01 (−0.15 to 0.18)	.88
Total other benefits	−0.01 (−1.22 to 1.20)	.99	−0.24 (−1.65 to 1.17)	.73
Total benefits	0.07 (−2.45 to 2.59)	.96	−0.05 (−2.75 to 2.65)	.97
Physical improvements and housing	0.02 (−0.03 to 0.07)	.47	0.02 (−0.03 to 0.07)	.47
Economic development	0.01 (−0.01 to 0.02)	.27	0.01 (−0.01 to 0.02)	.35
Community support	−0.01 (−0.03 to 0.01)	.25	−0.01 (−0.03 to 0.01)	.27
Environmental improvements	<0.01 (−0.01 to 0.00)	.71	<0.01 (−0.01 to 0.00)	.64
Leadership development and training for community members	<0.01 (−0.01 to 0.00)	.12	<0.01 (−0.01 to 0.00)	.11
Coalition building	<0.01 (−0.00 to 0.00)	.48	<0.01 (−0.00 to 0.00)	.51
Community health improvement advocacy	<0.01 (−0.02 to 0.02)	.84	<0.01 (−0.02 to 0.02)	.84
Workforce development	0.01 (−0.00 to 0.03)	.11	0.01 (−0.00 to 0.03)	.08
Other community building activities	0.01 (−0.01 to 0.02)	.40	0.01 (−0.01 to 0.02)	.37
Total community building activities	0.02 (−0.03 to 0.06)	.48	0.02 (−0.03 to 0.07)	.46

Abbreviation: DID, difference-in-differences.

^a^ Adjusted with county-level controls for population, proportion of male residents, proportion of residents aged 65 years or older, proportion of black residents, proportion of Hispanic residents, income per capita, government transfers per capita, employment per capita, and state and year fixed effects.

## Discussion

The Patient Protection and Affordable Care Act led to historic decreases in the number of uninsured individuals, primarily via Medicaid expansion. Consequently, in the years following Medicaid expansion, tax-exempt hospitals in expansion states reported significant decreases in uncompensated care. In this study, these decreases were not accompanied by increases in other types of community benefit spending, such as community health investment or community-building activities, but instead by increases in reported unreimbursed Medicaid expenses. We posit that the increased reports of unreimbursed Medicaid expenses are associated with the 3 following categories of patients: those previously uninsured, those previously untreated, and those previously privately insured. All 3 categories of care-seekers only apply to hospitals that reported higher allocated Medicaid costs than their states reimbursed. First, when states expanded Medicaid eligibility, adults within the expansion umbrella who would seek hospital care regardless of insurance status moved from the category of uninsured to Medicaid insured. Second, individuals who previously did not seek care because they were uninsured may now have had access to needed hospital services. Third, in some instances Medicaid expansion may have resulted in small employers terminating employment-based health insurance (and, therefore, the employers’ cost thereof) because the affected employees would now qualify for Medicaid. Overall, while hospitals in expansion states received some reimbursement for patients for whom they previously received no compensation, this additional reimbursement may have been offset by hospital spending among patients who previously did not present at the hospital or whose reimbursements from Medicaid were smaller than those from previously held private insurance. It is also possible that tax-exempt hospitals changed the way they accounted for Medicaid patient expenses, so that the reimbursement was not sufficient to offset those expenses.

Our findings contrast slightly with those of Young et al,^11^ who found no change in how tax-exempt hospitals reported the patient health care aspect of community benefit spending. The principal reason for the difference is illustrated in our figures. First, there was no hospital response to Medicaid expansion in the year of Medicaid expansion. As the data set of Young et al^11^ ended in 2014, the authors did not have access to the 2 years during which we found associations. Second, we applied a difference-in-differences regression design that focused explicitly on how trends in states that expanded Medicaid differed from states that had not yet expanded.

Our findings are largely consistent with those of Alberti et al,^9^ who analyzed Internal Revenue Service form 990 filings for 169 tax-exempt teaching hospitals. Like our study, they found statistically significant decreases in uncompensated care and increases in unreimbursed Medicaid among hospitals in states that expanded Medicaid vs those that did not. While they reported increases in total community benefit spending, this result was not statistically significant (*P* = .53).

### Limitations

We acknowledge several important limitations to this study. First, this study only documented changes in what hospitals reported on tax filings. Costs for charity care and unreimbursed Medicaid expenses were computed as total expenses scaled by the ratio of charges in those categories to total charges. This formula is theoretically susceptible to manipulation by hospitals. For example, if new patients seeking care as the result of Medicaid expansion made disproportionate use of certain services, those services could be assigned disproportionately higher charges relative to existing patients, thereby loading more overhead cost into the Medicaid category. There could even be changes in charges for services most frequently used by patients who obtained charity or Medicaid care. The worksheets that contain the calculations behind the reported filing numbers are not filed with the Internal Revenue Service; thus, we cannot account for these potential changes.

Second, our difference-in-differences approach relied on the assumption that trends in nonexpansion states were good counterfactuals for trends in Medicaid expansion states. While we could not test this assumption directly, we found no evidence of nonparallel trends in the period before expansion (Figure 2 and Figure 3).

Third, these cost measures represented an average cost accounting approach. Under this approach, an increased volume of Medicaid patients may appear to have negative consequences on a hospital’s ability to provide other types of community benefit. Policy decisions are best made at the margin, especially when hospitals may incur profits on additional volume even at lower reimbursement rates. Under a marginal costing approach, which is not what is required by the Internal Revenue Service, hospitals may be financially no worse off, or possibly better off, after Medicaid expansion.

Fourth, our analysis only represents the changes among hospitals in our sample. While we had tax filings for the universe of all tax-exempt hospitals, we were unable to match approximately 10% of these hospitals with control variables from American Hospital Association data. Unmatched hospitals spent more on charity care, and it is possible that increases in unreimbursed Medicaid expenses may have been larger or smaller than declines in charity care expenses associated with Medicaid expansion. Further, even if we had matched these hospitals with control variables, our results still would have been limited to tax-exempt hospitals, which are required to report these categories of charitable spending, rather than include the universe of hospitals.

## Conclusions

In this study, Medicaid expansion was associated with decreases in the amount of community benefits reported by tax-exempt hospitals as charity care. However, this decrease was offset by reported increases in the provision of unreimbursed Medicaid expense. Other categories of community benefit spending were largely unaffected. Overall, there was no association between Medicaid expansion and total community benefit spending.

## Notes

Supplement. **eTable 1. **Summary Characteristics for Hospitals Before Medicaid Expansion **eTable 2. **Associations Between Medicaid Expansion and Other Expenditures, Measured in Proportion of Hospital Expense
