# Supplementary material for: Association of Nonprofit Hospitals’ Charitable Activities With Unreimbursed Medicaid Care After Medicaid Expansion
Source: JAMA Netw Open. 2020 Feb 26;3(2):e200012. doi: 10.1001/jamanetworkopen.2020.0012 (PMC7137680; doi:10.1001/jamanetworkopen.2020.0012)
Supplement: Supplement. — eTable 1. Summary Characteristics for Hospitals Before Medicaid Expansion eTable 2. Associations Between Medicaid Expansion and Other Expenditures, Measured in Proportion of Hospital Expense [file jamanetwopen-3-e200012-s001.pdf]

Supplementary Online Content

Stoecker C, Demosthenidy M, Shao Y, Long H. Association of nonprofit hospitals' charitable activities with unreimbursed Medicaid care after Medicaid expansion. *JAMA Netw Open*. 2020;3(2):e200012. doi:10.1001/jamanetworkopen.2020.0012

eTable 1. Summary Characteristics for Hospitals Before Medicaid Expansion

eTable 2. Associations Between Medicaid Expansion and Other Expenditures, Measured in Proportion of Hospital Expense

This supplementary material has been provided by the authors to give readers additional information about their work.

© 2020 Stoecker C et al. *JAMA Network Open.*

eTable 1. Summary Characteristics for Hospitals Before Medicaid Expansion

Pre-years for hospitals with a post-year (year<=2013)						
	Matched hospitals		Unmatched hospitals			
	Hospitals in never expanded states	Hospitals in ever expanded states	Hospitals in never expanded states	Hospitals in ever expanded states	p-value (1)	p-value of (2)
Total community benefit at cost ($)	17,382.19	21,803.23	48,677.40	88,979.18	0.00	0.00
	(46,488.33)	(47,290.59)	(112,825.80)	(204,405.59)		
Total community benefit at cost (%)	0.08	0.08	0.10	0.09	0.00	0.00
	(0.08)	(0.07)	(0.15)	(0.10)		
Financial assistance at cost ($)	6,076.71	4,112.45	19,847.46	18,537.59	0.00	0.00
	(11,898.97)	(6,729.36)	(48,450.43)	(34,610.40)		
Financial assistance at cost (%)	0.14	0.18	0.12	0.15	0.01	0.17
	(0.24)	(0.28)	(0.22)	(0.25)		
Unreimbursed Medicaid ($)	4,301.39	7,867.20	9,011.19	27,323.73	0.00	0.00
	(10,422.28)	(19,088.57)	(26,050.34)	(59,543.25)		
Unreimbursed Medicaid (%)	0.11	0.09	0.11	0.08	0.47	0.94
	(0.21)	(0.18)	(0.22)	(0.17)		
Costs of other means-tested government programs ($)	379.97	533.55	717.25	1,812.20	0.00	0.24

© 2020 Stoecker C et al. *JAMA Network Open.*

	(4,033.50)	(3,060.48)	(3,323.68)	(7,841.12)		
Costs of other means-tested government programs (%)	0.05	0.05	0.05	0.03	0.13	0.83
	(0.15)	(0.16)	(0.15)	(0.15)		
Total charity care ($)	10,752.61	12,497.80	29,575.91	47,684.70	0.00	0.00
	(20,007.29)	(23,284.96)	(63,681.17)	(94,544.36)		
Total charity care (%)	0.10	0.09	0.10	0.10	0.36	0.54
	(0.15)	(0.14)	(0.16)	(0.16)		
Community health improvement services and community benefit operations ($)	645.32	1,008.14	3,157.70	3,201.58	0.00	0.00
	(2,049.95)	(2,806.85)	(15,473.45)	(9,549.26)		
Community health improvement services and community benefit operations (%)	0.19	0.20	0.16	0.17	0.02	0.11
	(0.23)	(0.24)	(0.24)	(0.22)		
Health professions education ($)	2,028.80	3,847.94	5,121.42	12,558.50	0.00	0.00
	(11,603.70)	(14,431.35)	(16,633.83)	(31,123.83)		
Health professions education (%)	0.13	0.13	0.14	0.17	0.00	0.41
	(0.21)	(0.22)	(0.25)	(0.26)		

© 2020 Stoecker C et al. *JAMA Network Open.*

Subsidized health services ($)	1,624.22	2,079.61	2,764.34	4,545.54	0.00	0.02
	(6,129.19)	(5,918.98)	(12,239.08)	(10,082.15)		
Subsidized health services (%)	0.08	0.10	0.07	0.10	0.89	0.58
	(0.18)	(0.21)	(0.17)	(0.19)		
Research ($)	1,462.22	1,777.03	6,930.09	16,477.69	0.00	0.00
	(20,359.24)	(16,480.23)	(49,317.82)	(109,471.56)		
Research (%)	0.02	0.03	0.04	0.06	0.00	0.02
	(0.08)	(0.11)	(0.12)	(0.15)		
Cash and in-kind contributions to community groups ($)	755.52	360.22	973.12	2,455.52	0.00	0.30
	(3,887.32)	(1,534.94)	(3,208.10)	(12,564.66)		
Cash and in-kind contributions to community groups (%)	0.09	0.09	0.08	0.10	0.38	0.44
	(0.16)	(0.16)	(0.15)	(0.16)		
Total other benefits ($)	6,452.57	9,109.40	18,795.21	40,838.23	0.00	0.00
	(35,319.32)	(29,349.85)	(67,104.59)	(143,993.30)		
Total other benefits (%)	0.18	0.17	0.12	0.12	0.00	0.00
	(0.26)	(0.26)	(0.22)	(0.21)		
Total benefits ($)	17,131.09	21,588.49	48,373.63	89,785.39	0.00	0.00
	(46,166.43)	(47,385.71)	(112,083.84)	(206,029.66)		

© 2020 Stoecker C et al. *JAMA Network Open.*

Total benefits (%)	0.10	0.10	0.11	0.11	0.22	0.12
	(0.11)	(0.10)	(0.15)	(0.13)		
Physical improvements and housing ($)	45.23	10.19	2.15	72.23	0.00	0.37
	(725.94)	(104.06)	(12.10)	(633.00)		
Physical improvements and housing (%)	0.00	0.00	0.00	0.00	0.63	0.61
	(0.03)	(0.02)	(0.06)	(0.02)		
Economic development ($)	3.39	28.64	23.18	13.61	0.59	0.00
	(24.91)	(591.21)	(232.51)	(95.07)		
Economic development (%)	0.00	0.00	0.00	0.00	0.55	0.22
	(0.01)	(0.03)	(0.01)	(0.02)		
Community support($)	33.76	40.21	55.30	111.33	0.00	0.41
	(371.75)	(190.23)	(288.27)	(643.18)		
Community support(%)	0.01	0.02	0.01	0.02	0.52	1.00
	(0.04)	(0.07)	(0.04)	(0.07)		
Environmental improvements ($)	1.08	5.24	25.39	2.45	0.41	0.01
	(12.46)	(61.72)	(356.60)	(12.54)		
Environmental improvements (%)	0.00	0.00	0.00	0.00	0.06	0.62
	(0.01)	(0.02)	(0.01)	0.00		

© 2020 Stoecker C et al. *JAMA Network Open.*

Leadership development and training for community members ($)	2.42	5.39	1.00	8.49	0.38	0.54
	(35.29)	(61.33)	(5.10)	(61.97)		
Leadership development and training for community members (%)	0.00	0.00	0.00	0.00	0.96	0.23
	(0.01)	(0.03)	0.00	(0.04)		
Coalition building ($)	7.50	8.02	8.60	79.39	0.00	0.82
	(67.28)	(44.39)	(29.60)	(394.94)		
Coalition building (%)	0.00	0.01	0.00	0.01	0.92	0.78
	(0.02)	(0.03)	(0.02)	(0.03)		
Community health improvement advocacy ($)	24.13	38.15	141.50	56.36	0.42	0.01
	(323.09)	(395.37)	(1,378.39)	(332.90)		
Community health improvement advocacy (%)	0.01	0.01	0.01	0.01	0.37	0.47
	(0.04)	(0.06)	(0.07)	(0.06)		
Workforce development ($)	50.12	49.76	40.12	98.85	0.03	0.53
	(281.40)	(379.16)	(187.17)	(406.70)		
Workforce development (%)	0.03	0.02	0.01	0.01	0.05	0.01
	(0.11)	(0.09)	(0.04)	(0.06)		

© 2020 Stoecker C et al. *JAMA Network Open.*

Other community building activities ($)	9.39	24.47	9.06	13.17	0.59	0.94
	(78.96)	(387.24)	(72.95)	(73.37)		
Other community building activities (%)	0.01	0.01	0.00	0.01	0.40	0.06
	(0.04)	(0.05)	(0.01)	(0.07)		
Total community building activities ($)	177.01	196.03	306.29	456.24	0.00	0.10
	(955.64)	(945.84)	(1,604.78)	(1,782.62)		
Total Community building activities (%)	0.06	0.07	0.04	0.04	0.00	0.07
	(0.15)	(0.15)	(0.12)	(0.13)		
Total expense ($)	176,783.15	225,506.41	472,704.61	941,980.15	0.00	0.00
	(273,805.30)	(364,872.93)	(844,499.59)	(1,994,693.74)		
Total revenue ($)	189,838.77	237,242.24	505,943.13	1,004,229.76	0.00	0.00
	(297,633.86)	(386,225.26)	(905,292.91)	(2,153,019.46)		
Profit margin (hospital)	0.02	0.03	0.04	0.02	0.58	0.46
	(0.44)	(0.22)	(0.15)	(0.23)		
Male (%) (county)	0.49	0.49				
	(0.02)	(0.01)				

© 2020 Stoecker C et al. *JAMA Network Open.*

Age over 65 years old (county)	0.16	0.16				
	(0.05)	(0.04)				
Black (%) (county)	0.14	0.10				
	(0.16)	(0.12)				
Hispanic (%) (county)	0.10	0.10				
	(0.13)	(0.12)				
Population (county)	320,114.85	672,494.31				
	(590,468.26)	(1,493,909.50)				
Income per capita ($) (county)	39,907.30	44,613.60				
	(9,356.60)	(15,596.05)				
Transfers per capita (county)	7.88	8.37				
	(1.60)	(1.49)				
Employment per capita (county)	0.59	0.58				
	(0.14)	(0.19)				
Number of contract managed hospitals	90	80				
Number of teaching hospitals	35	110				

© 2020 Stoecker C et al. *JAMA Network Open.*

Number of hospitals with beds > median	218	470				
Number of rural hospitals	178	241				
Number of hospitals with high margin	408	686				
Number of independent hospitals	33	102				
Number of states	19	32	17	29		
Number of hospitals	718	1,331	118	178		
Number of observations	1,396	2,590	232	345		

Notes: Table show s outcome variables (measured in dollars and in proportion of hospital expense) and characteristics of hospitals by the end of 2013, by Medicaid expansion status. Column 1 and column 3 show the characteristics of hospitals in states which did not expand Medicaid by the end of 2016. Column 2 and column 4 show the characteristics of hospitals in states w hich expanded Medicaid by the end of 2016. Column 1 and 2 show the characteristics of hospitals whose Schedule H findings w ere matched. Column 3 and 4 show the characteristics of hospitals whose Schedule H findings w ere not matched. P-value (1) compares the difference between matched and unmatched hospitals in non-expanded sates. P-value (2) compares the difference of between matched and unmatched hospitals in expanded sates. All statistics in the table are the mean at the hospital-fiscal year level. Standard deviations are show n in parenthesis.

© 2020 Stoecker C et al. *JAMA Network Open.*

eTable 2. Associations Between Medicaid Expansion and Other Expenditures, Measured in Proportion of Hospital Expense

	DID		DID with controls	
Financial assistance at cost (%)	-0.02	[-0.06,0.02]	-0.02	[-0.06,0.02]
	0.35		0.38	
Unreimbursed Medicaid (%)	0.02	[0.01,0.04]	0.02	[0.01,0.04]
	0.01		0.01	
Costs of other means-tested government programs (%)	0	[-0.02,0.02]	0	[-0.02,0.02]
	0.9		0.97	
Total charity care (%)	0.01	[-0.01,0.02]	0.01	[-0.01,0.02]
	0.46		0.47	
Community health improvement services and community benefit operations (%)	-0.01	[-0.04,0.02]	-0.01	[-0.04,0.02]
	0.38		0.43	
Health professions education (%)	-0.01	[-0.03,0.01]	-0.01	[-0.03,0.02]
	0.54		0.61	
Subsidized health services (%)	-0.01	[-0.03,0.01]	-0.01	[-0.03,0.01]
	0.29		0.28	
Research (%)	0	[-0.01,0.00]	0	[-0.01,0.01]
	0.36		0.41	
Cash and in-kind contributions to community groups (%)	0	[-0.02,0.02]	0	[-0.02,0.02]
	0.85		0.92	
Total other benefits (%)	-0.01	[-0.03,0.02]	0	[-0.03,0.02]
	0.67		0.7	
Total benefits (%)	0	[-0.01,0.01]	0	[-0.01,0.01]
	0.59		0.58	

© 2020 Stoecker C et al. *JAMA Network Open.*

Physical improvements and housing (%)	0	[-0.00,0.00]	0	[-0.00,0.00]
	0.88		0.91	
Economic development (%)	0	[-0.00,0.00]	0	[-0.00,0.00]
	0.57		0.6	
Community support (%)	0	[-0.01,-0.00]	0	[-0.01,-0.00]
	0.02		0.02	
Environmental improvements (%)	0	[-0.00,0.00]	0	[-0.00,0.00]
	0.1		0.08	
Leadership development and training for community members (%)	0	[-0.00,-0.00]	0	[-0.00,-0.00]
	0.04		0.04	
Coalition building (%)	0	[-0.00,0.00]	0	[-0.00,0.00]
	0.07		0.07	
Community health improvement advocacy (%)	0	[-0.01,0.00]	0	[-0.01,0.00]
	0.3		0.28	
Workforce development (%)	0.01	[-0.00,0.02]	0.01	[-0.00,0.02]
	0.13		0.15	
Other community building activities (%)	0	[-0.00,0.00]	0	[-0.00,0.00]
	0.4		0.39	
Total Community building activities (%)	0	[-0.02,0.01]	-0.01	[-0.02,0.01]
	0.52		0.48	
Total community benefit at cost (%)	0	[-0.00,0.01]	0	[-0.00,0.01]
	0.63		0.61	
Observations	10,154		10,154	

Notes: The first two columns are from a basic differences-in-differences (DID) model and the last two columns include county-level controls for population, proportion that are female, age 65 or older, black, Hispanic, income per capita, government transfers per capita, and employment per capita. Outcome data comes from hospital reports of charitable activities on tax filings for 2012-2016. Coefficients and p-value under each coefficient are listed in the first and third columns. 95% confidence intervals with brackets are listed in the second and fourth column.

© 2020 Stoecker C et al. *JAMA Network Open.*
